# Cultivable microbial community in 2-km-deep, 20-million-year-old subseafloor coalbeds through ~1000 days anaerobic bioreactor cultivation

**DOI:** 10.1038/s41598-019-38754-w

**Published:** 2019-02-19

**Authors:** Hiroyuki Imachi, Eiji Tasumi, Yoshihiro Takaki, Tatsuhiko Hoshino, Florence Schubotz, Shuchai Gan, Tzu-Hsuan Tu, Yumi Saito, Yuko Yamanaka, Akira Ijiri, Yohei Matsui, Masayuki Miyazaki, Yuki Morono, Ken Takai, Kai-Uwe Hinrichs, Fumio Inagaki

**Affiliations:** 1 Department of Subsurface Geobiological Analysis and Research (D-SUGAR), Japan Agency for Marine-Earth Science and Technology (JAMSTEC), Yokosuka, Kanagawa 237-0061 Japan; 2 Research and Development Center for Submarine Resources, JAMSTEC, Yokosuka, Kanagawa 237-0061 Japan; 3 Project Team for Development of New-generation Research Protocol for Submarine Resources, JAMSTEC, Yokosuka, Kanagawa 237-0061 Japan; 4 Kochi Institute for Core Sample Research, JAMSTEC, Nankoku, Kochi 783-8502 Japan; 5 MARUM Center for Marine Environmental Sciences and Department of Geosciences, University of Bremen, D-28359 Bremen, Germany; 6 Institute of Oceanography, National Taiwan University, Taipei, 106 Taiwan; 7 Research and Development Center for Ocean Drilling Science, JAMSTEC, Yokohama, Kanagawa 236-0001 Japan

## Abstract

Recent explorations of scientific ocean drilling have revealed the presence of microbial communities persisting in sediments down to ~2.5 km below the ocean floor. However, our knowledge of these microbial populations in the deep subseafloor sedimentary biosphere remains limited. Here, we present a cultivation experiment of 2-km-deep subseafloor microbial communities in 20-million-year-old lignite coalbeds using a continuous-flow bioreactor operating at 40 °C for 1029 days with lignite particles as the major energy source. Chemical monitoring of effluent samples via fluorescence emission-excitation matrices spectroscopy and stable isotope analyses traced the transformation of coalbed-derived organic matter in the dissolved phase. Hereby, the production of acetate and ^13^C-depleted methane together with the increase and transformation of high molecular weight humics point to an active lignite-degrading methanogenic community present within the bioreactor. Electron microscopy revealed abundant microbial cells growing on the surface of lignite particles. Small subunit rRNA gene sequence analysis revealed that diverse microorganisms grew in the bioreactor (e.g., phyla *Proteobacteria*, *Firmicutes*, *Chloroflexi*, *Actinobacteria*, *Bacteroidetes*, *Spirochaetes*, *Tenericutes*, *Ignavibacteriae*, and SBR1093). These results indicate that activation and adaptive growth of 2-km-deep microbes was successfully accomplished using a continuous-flow bioreactor, which lays the groundwork to explore networks of microbial communities of the deep biosphere and their physiologies.

## Introduction

Over the past two decades, scientific ocean drilling has demonstrated that numerous microbes exist in the global deep subseafloor sediment, from the continental margins to open ocean gyres, comprising approximately 10^29^ microbial cells and 4 Pg of biomass carbon on our planet^1,2^. Porewater geochemistry suggests that organic matter-fueled microbial energy respiratory activity is extraordinary low, ranging from 2.8 × 10^−18^ to 1.1 × 10^−14^ moles/e^−^/cell/year between the anoxic eastern equatorial Pacific and the oxic South Pacific Gyre sediments, respectively^3–5^. Culture-independent molecular ecological studies (e.g., PCR-mediated 16S rRNA and functional gene analysis, or metagenomics) of the above-mentioned subseafloor settings showed that they harbor diverse microbial communities, most of which are phylogenetically distinct from those living in the Earth’s surface environments^6–9^; hence, their physiology and metabolic functions still remain largely unknown^10,11^.

To gain insight into deep subseafloor microbial life, cultivation is crucial. Previous cultivation efforts on sediment core samples, however, indicated a high resistance of deeply buried microbial communities to conventional batch-type cultivation techniques. Consequently, only a small fraction of indigenous deep microbes could be isolated thus far from ≥10 m below seafloor (mbsf) sediment samples, whose members are primarily affiliated with the phyla *Proteobacteria*, *Firmicutes*, *Actinobacteria*, and *Bacteroidetes* or *Euryarchaeota* genera *Methanoculleus*, *Methanococcus*, and *Methanosarcina*^8,12–14^. Nevertheless, stable isotope tracer incubation experiments combined with nanometer-scale secondary ion mass spectrometry (NanoSIMS) analysis confirmed that more than 70% of the total microbial cells are viable, despite having very slow biomass turnover rates^15,16^. Thus, cultivation of deep subseafloor microbes through batch-type techniques may be impeded by their extraordinarily low metabolic activity under energy-limited conditions^5^ and/or the “substrate-accelerated death” phenomenon, wherein microbial cells are damaged when suddenly exposed to high substrate concentrations in rich laboratory media^17^.

Given the limited success of previous efforts to cultivate deep subseafloor microbes, new cultivation approaches are needed. Parkes *et al*.^18^ applied a high-pressure anaerobic enrichment system (i.e., DeepIsoBUG) for gas hydrate-bearing sediments and successfully obtained some anaerobic bacteria (e.g., genera *Acetobacterium* and *Clostridium*). Imachi and co-workers (2011, 2014, 2017)^19–21^ applied a continuous-flow bioreactor cultivation technique to overcome the limitation of batch-type cultivation and successfully enriched previously uncultured lineages from deep subseafloor sediments. These studies employed a down-flow hanging sponge (DHS) reactor system, which was originally developed for treating municipal sewage in developing countries at a low cost^22^. Specifically, a polyurethane sponge used in the DHS reactor ensures medium pore space to provide a larger surface area for microbial colonization and extended cell residence time. Such continuous-flow bioreactor cultivation can maintain the low concentrations of substrates found in the natural environments and outflow the accumulated metabolic products that may inhibit microbial growth. These continuous-flow reactors thereby might increase the culturability of subseafloor microorganisms in a controlled manner and serve as better sources (incubators) for the isolation of microorganisms than the original samples.

Recently, using a DHS reactor, Inagaki *et al*.^23^ established a methanogenic enrichment culture from ~2-km-deep subseafloor coalbed samples obtained using the riser-drilling technology of the deep-sea drilling vessel *Chikyu* during the Integrated Ocean Drilling Program (IODP) Expedition 337. In this study, we report the extensive microbiological and biogeochemical investigations over 1000 days of DHS reactor operation, including the detailed cultivation procedure, microbial community structure, and microbial metabolism during the course of the bioreactor operation. We observed that phylogenetically diverse indigenous microbial populations were cultivated in the bioreactor. The cultivars seemingly grow on and transform coalbed-derived organic matter. Additionally, three anaerobic microorganisms, including a methanogenic archaeon, were obtained in pure culture from the bioreactor enrichment culture.

## Results

### Microbial metabolism during enrichment in the DHS bioreactor

**Figure 1:**
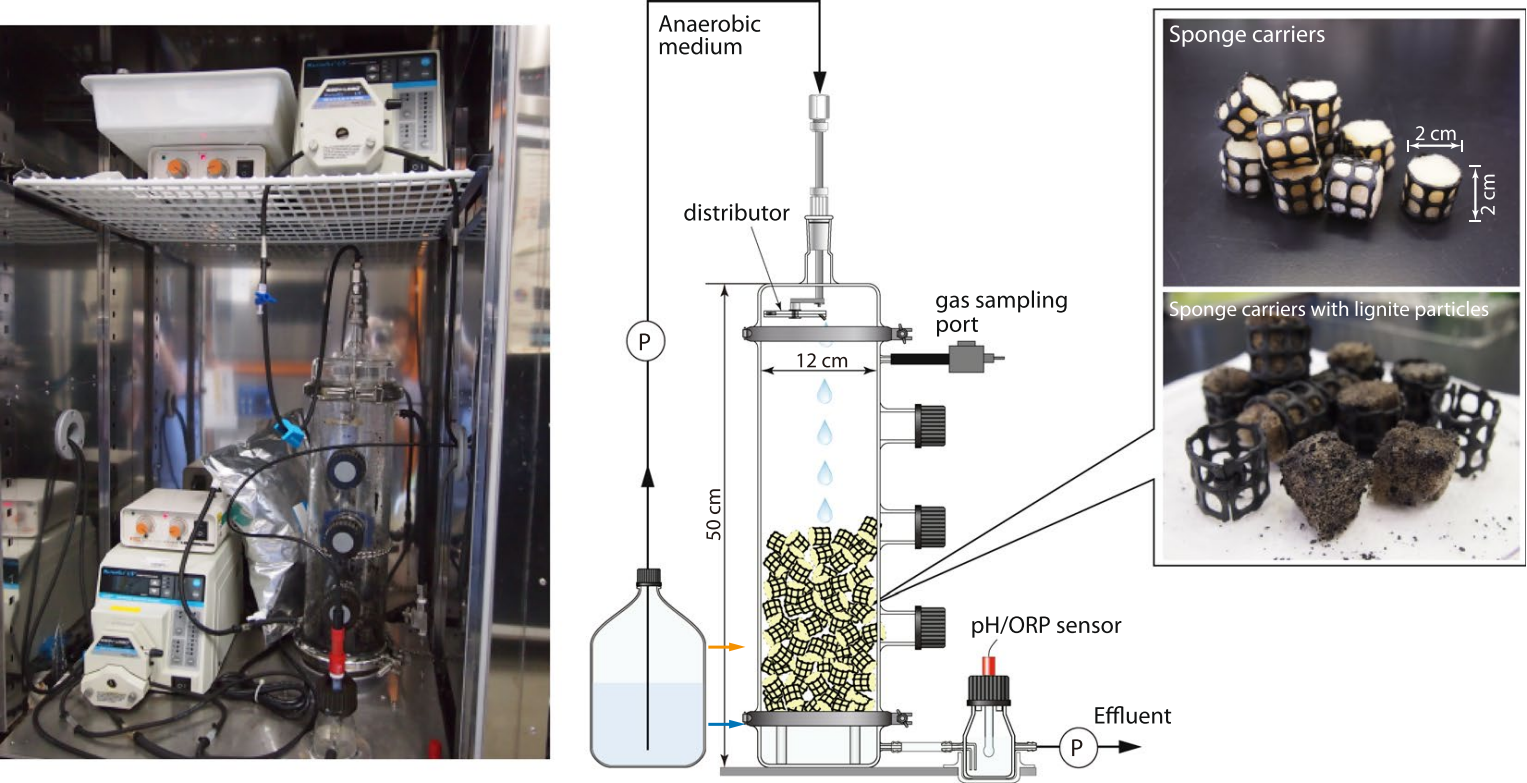
Photographs and schematic diagram of the DHS bioreactor system used in this study. The photographs on the right-hand side show virgin sponge carriers (upper) and lignite coal particles attached to the sponge carriers (lower). The black particles on the sponge carriers are lignite coal. Blue arrow on the schematic diagram indicates normal water level. Orange arrow shows the water level at 805 days, which was caused by the effluent pump failure.

DHS reactor (Fig. 1) operation at a near *in situ* temperature of 40 °C over the course of 1029 days yielded effluent at the mean oxidation-reduction potential (ORP) value of −430 ± 47 mV (n = 478), indicating strict maintenance of anaerobic conditions. The mean pH of the effluent was 7.24 ± 0.16 (Supplementary Fig. S1).

**Figure 2:**
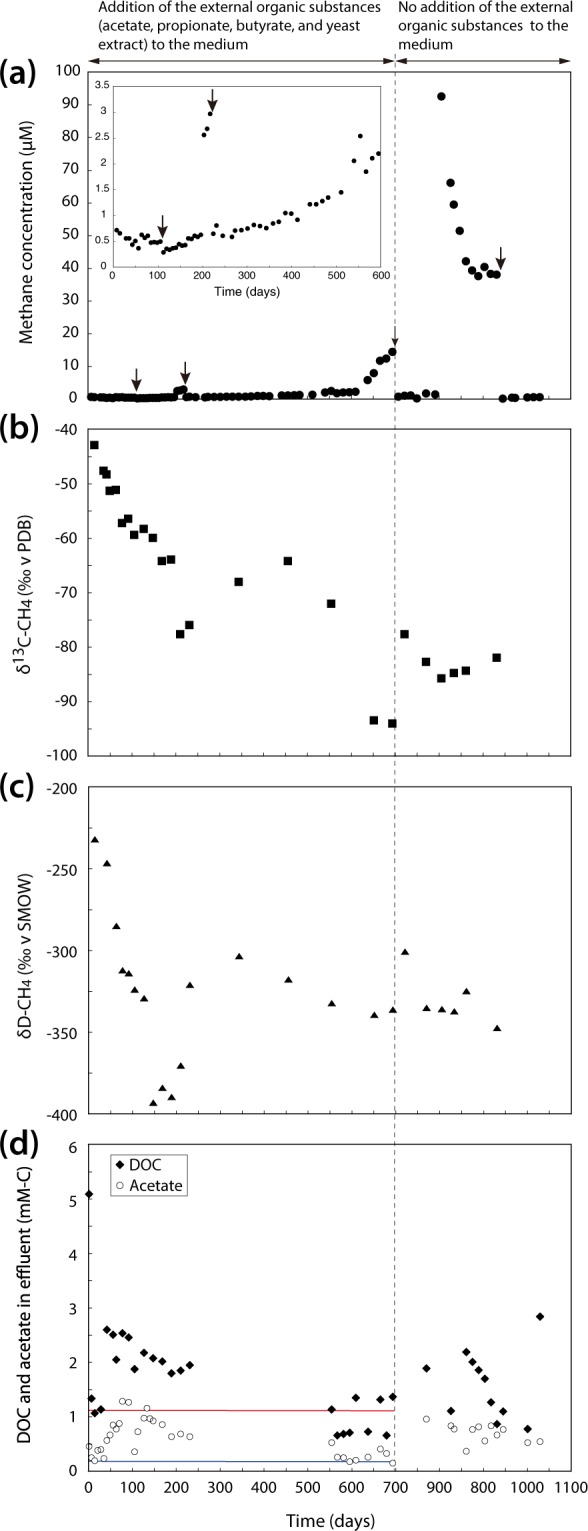
Changes of substrate and product concentrations during the DHS reactor operation. (**a**) Methane concentrations in the headspace of the DHS bioreactor. Arrows indicate the sampling days for sponge carriers from the bioreactor. On each sampling days, accumulated methane became zero because the lid of the bioreactor was opened and nitrogen gas was used for flushing during the sampling. Methane concentration became zero on day 753 day, because the lid of bioreactor glass column was opened and the inside of the medium inlet was washed to remove mineral precipitate that had caused a clog. (**b**) δ^13^C-CH_4_ values (‰ versus the Vienna Pee Dee Belemnite (VPDB)). (**c**) δD-CH_4_ values (‰ versus the Standard Mean Ocean Water (SMOW)). (**d**) DOC and acetate concentrations in the effluent. Blue and red lines shows theoretical DOC and acetate concentrations in the influent (i.e., 1.13 and 0.14 mM-C) until 693 days, respectively. Dotted line marks the change in substrate addition to the DHS reactor.

As an indicator of microbial metabolic activity, methane in the reactor headspace was monitored continuously during the entire period of reactor operation after the first measurement at 7 days (Fig. 1a, Supplementary Table S1). At the early stage (0–105 days), methane concentration in the headspace of the DHS reactor decreased from 0.7 to 0.4 µM in the first 56 days then remained around 0.5 µM. Additionally, δ^13^C and δD values of methane decreased sharply from −42.9 to −59.4‰ and from −232.1 to −323.9‰, respectively (Fig. 2b,c). The ^13^C-depleted methane suggests that methane absorbed on lignite particles, originally established in the subseafloor coalbed layers, was gradually replaced by newly produced methane from microorganisms in the DHS bioreactor because the latter δ^13^C and δD values of methane tend to be isotopically light^24^. Methane concentration gradually increased with operation time after 105 days. Notably, continuous methane-production was also observed even without the addition of external organic substances (i.e., acetate, propionate, butyrate, and yeast extract) into the medium after 694 days (Fig. 2a). At 805 days, methane concentration drastically increased to 92.6 µM, potentially as a consequence of accidental water level elevation in the bioreactor glass column owing to the effluent pump failure (see arrows in Fig. 1), thereby stimulating microbial metabolism. Following pump repair and restoration to the normal water level on day 805, the methane concentration decreased and remained around 40 µM between 861 and 931 days. Constant methane production was observed after sampling at 932 days, with the concentration increasing from 0.21 to 0.62 µM. Methane δ^13^C and δD values gradually decreased with operation time after 105 days, although the values fluctuated (Fig. 2b,c). The lowest methane isotopic composition δ^13^C and δD values were −94.2 and −393.0‰, respectively, indicating that microbial methanogenesis occurred in the DHS reactor.

Dissolved organic carbon (DOC) concentrations were generally higher in the effluent than the influent (Fig. 2d, Supplementary Table S1), indicating the release of lignite-derived organics into the aqueous phase. Acetate was detected in all effluent samples, at concentrations higher than what was initially added to the bioreactor, except at 595 and 693 days. The δ^13^C values of effluent acetate were between −23.6 and −41.8‰ (Supplementary Table S1), suggesting production via microbial fermentation and possibly acetogenesis^25^. Concentrations of butyrate and propionate that were introduced to the reactor for the first 694 days were lower in the effluent than the influent with a few exceptions (Supplementary Table S1), suggesting their utilization by microbial metabolism within 70 h of hydraulic retention time (HRT) in the reactor. Effluent propionate, but not butyrate, was detected even after stopping the supply to the reactor. Effluent propionate concentrations gradually decreased along with operation to below the detection limit after 945 days of operation, suggesting propionate was a lignite-degradation intermediate in the reactor. A tiny amount of formate was also produced during the reactor cultivation (Supplementary Table S1).

**Figure 3:**
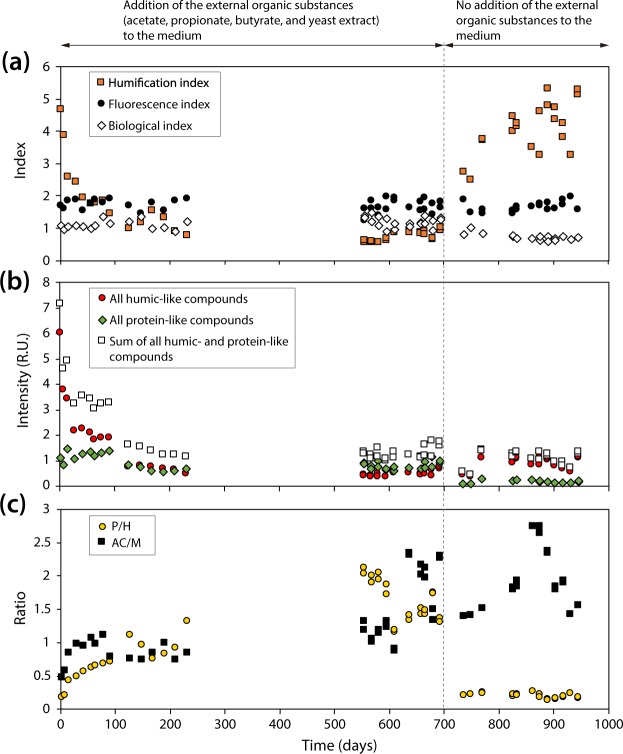
Changes in fluorescent dissolved organic matter (DOM) in effluent samples of the DHS reactor. (**a**) HIX – Humification index, FI – fluorescence index and BIX – biological index. The constant values of the fluorescence index (ca. 1.8) and biological index (ca. 1) indicate microbially-derived organic matter that is freshly produces and increases in the humification index indicate ongoing humification of the DOM. (**b**) Relative intensities (R.U. – raman unit) of humic-like and protein-like compounds over time. (**c**) Ratios of observed fluorescence peaks, P/H (protein-like over humic-like) and AC/M (conjugation degree of humic-like substances). Fluorescent peak assignments are according to Fellman *et al*.^26^.

Fluorescence spectroscopic characterization of the dissolved organic matter (DOM) in the effluent indicated that the DOM was mainly microbially derived (with a fluorescence index [FI] around 1.8) and recently produced (with a biological index [BIX] of 1) (Fig. 3a). Excitation-emission matrices (EEM) fluorescence spectroscopy analysis revealed three distinct phases of microbial activity associated with lignite-degradation over the period of reactor operation (Fig. 3). In the first phase (0 to approximately 100 days), humic-like compounds were abundantly present but decreased sharply. We expect these humic-like compounds to be comprised of irregular complexes of polysaccharide and proteinaceous material and phenolic compounds, which are difficult to define structurally^26^. Expected degradation products are plant lignin derived phenols^27^, but other lignite-derived structures such as benzene, polycyclic aromatic hydrocarbon or furan derivatives are also likely present. The low AC/M ratio (conjugation degree of humic-like substances) points to the presence of low molecular weight humics that are typically associated with microbial activity^28^. Size and chemical structure of these compounds were not further defined, but previous studies identified these low molecular weight compounds to be on average 665 Da in size^29^. We conclude that the low AM/C ratio could reflect the release of humic-like compounds adsorbed on lignite and/or microbial degradation. The second phase (100 to 721 days) was characterized by an overall loss of humic-like peaks and a relative domination of protein-like compounds (protein-like over humic-like [P/H] > 1). This change in fluorescent DOM was most likely derived from an increase in the activity of the microbial community and/or the added yeast extract in the effluent medium. The third phase (821 to 875 days) was dominated by a notable increase in humification of the DOM (humification index > 3), where humic-like, conjugated (aromatic) high molecular weight compounds increased. Size and chemical structure of these high molecular weight compounds were not determined in this study, but are assumed to be >1000 Da in size^29^ and assumed to contain either 3 to 5 (C peak) or 7 (A peak) fused aromatic rings^30^. The rise in the humification index occurred after the addition of organic substrates to the reactor was stopped and we interpret it to be caused by the release of highly humified organic matter via microbial degradation of lignite. Notably, the humification signal coincides with the highest AC/M peak after 821 days and a subsequent decrease after 875 days to levels observed in the second phase. The decrease in the AC/M ratio can be assigned to a decrease of terrestrial humic-like compounds, such as plant lignin derived phenols, indicating their consumption during the release of highly humified organic matter. The release of high molecular weight aromatics into the water phase and their subsequent degradation indicated an ongoing activation and potential utilization of lignite components by the enriched microorganisms. These results suggest that a shift in organic matter utilization of the reactor community occurred after the addition of external organic substances was stopped (after 694 days).

### Microscopic observation of the enriched microbial community

**Figure 4:**
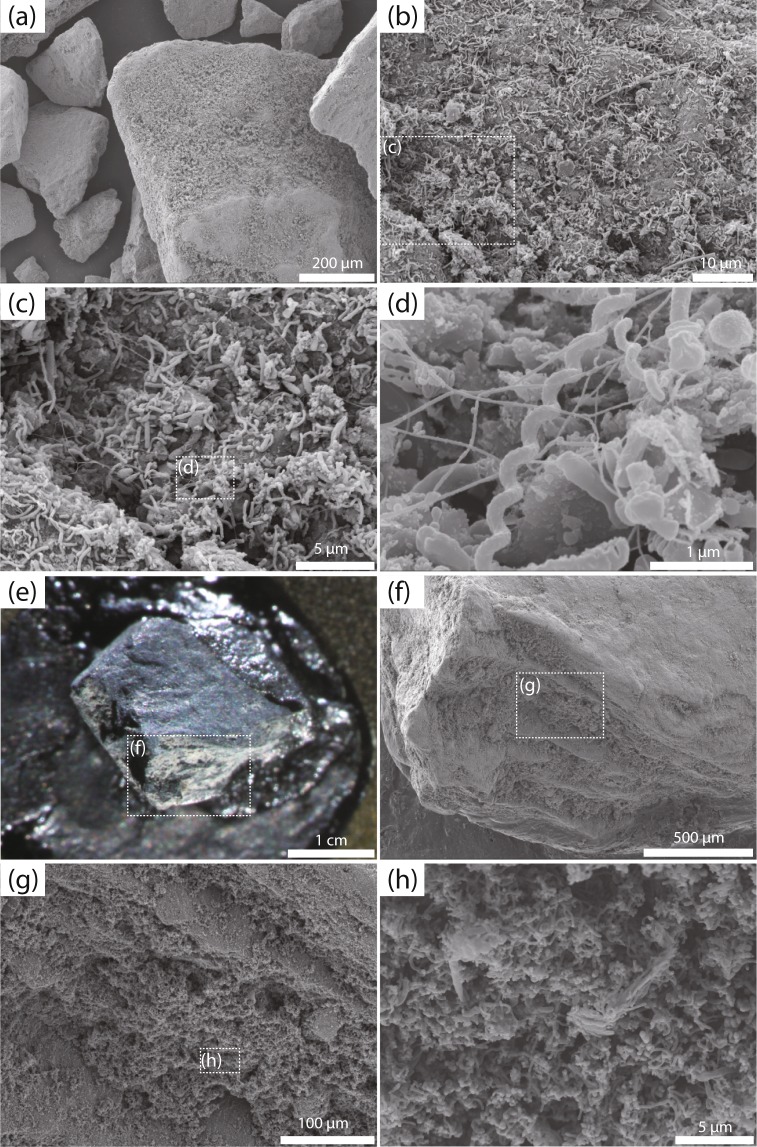
Scanning electron and stereo microscopic images of lignite particles incubated in the DHS bioreactor. All the lignite particles were attached to the sponge carriers. (**a**–**d**) Scanning electron microscopic (SEM) images of lignite particles and attached microorganisms collected at 694 days of reactor operation. (**e**) Stereo micrograph of a lignite particle having a white microbial mat, which was collected at 932 days. (**f**–**h**) High-magnification SEM images of the lignite particle (**e**). White dotted-line squares indicate high-magnification areas.

To ascertain microbial growth in the bioreactor, we microscopically examined the effluent collected at 29 and 107 days. Microscopic observation revealed the occurrence of morphologically diverse microorganisms in the effluent including F_420_-autofluorescent methanogens, rod-shaped cells and spherical spore-like particles^23^. The lignite particles attached on sponge carriers, which were collected at 694 and 932 days, were also observed by scanning electron microscopy (SEM). Notably, an abundance of microbial cells was observed on the lignite particle surfaces (Fig. 4). The cell morphology was diverse; e.g., spiral, straight rod, curved rod, and small cocci. Some rod-shaped microbes exhibited a long fiber structure (Fig. 4b–d). A white color biofilm was also observed on some 932-day lignite particles (Fig. 4e–h). Conversely, few microbial cells resided on sponge carrier surfaces (694 days, Supplementary Fig. S2a–c). Thus, microorganisms appeared to colonize exclusively on lignite particle surfaces in the DHS bioreactor system. In comparison, no microbial cells were observed on lignite particles before cultivation (Supplementary Fig. S2d–f).

### Biomass-estimate in the DHS reactor enrichment

Increases in the amount of DNA extracted from the enriched samples suggested significant microbial biomass development in the DHS reactor (Supplementary Table S2). To estimate how biomass developed during cultivation, we calculated cell numbers based on the amount of extracted DNA, assuming all microbial cells possessed 3 Mbp genomic DNA and an average nucleotide molecular weight of 330. The total number of cells ranged from 1.19 × 10^8^ to 5.02 × 10^9^ cells on 1 g enriched wet lignite sample (i.e., all the sponge carrier samples) (Supplementary Table S2). The estimated total cell numbers were 1–2 orders of magnitude higher in the lignite samples than in the effluent samples. Assuming an inoculum cell density of 2.89 × 10^4^ cells/g wet-weight (Supplementary Table S3), the number of cells on the lignite particles increased by roughly 3–5 orders of magnitude (ca. 4000–170000-fold) during the reactor cultivation. The wide range of cell numbers in the enriched lignite samples might result from uneven microbial colonization on the lignite particle surface, as shown in the SEM images (Fig. 4).

### Microbial community composition in the DHS bioreactor

**Figure 5:**
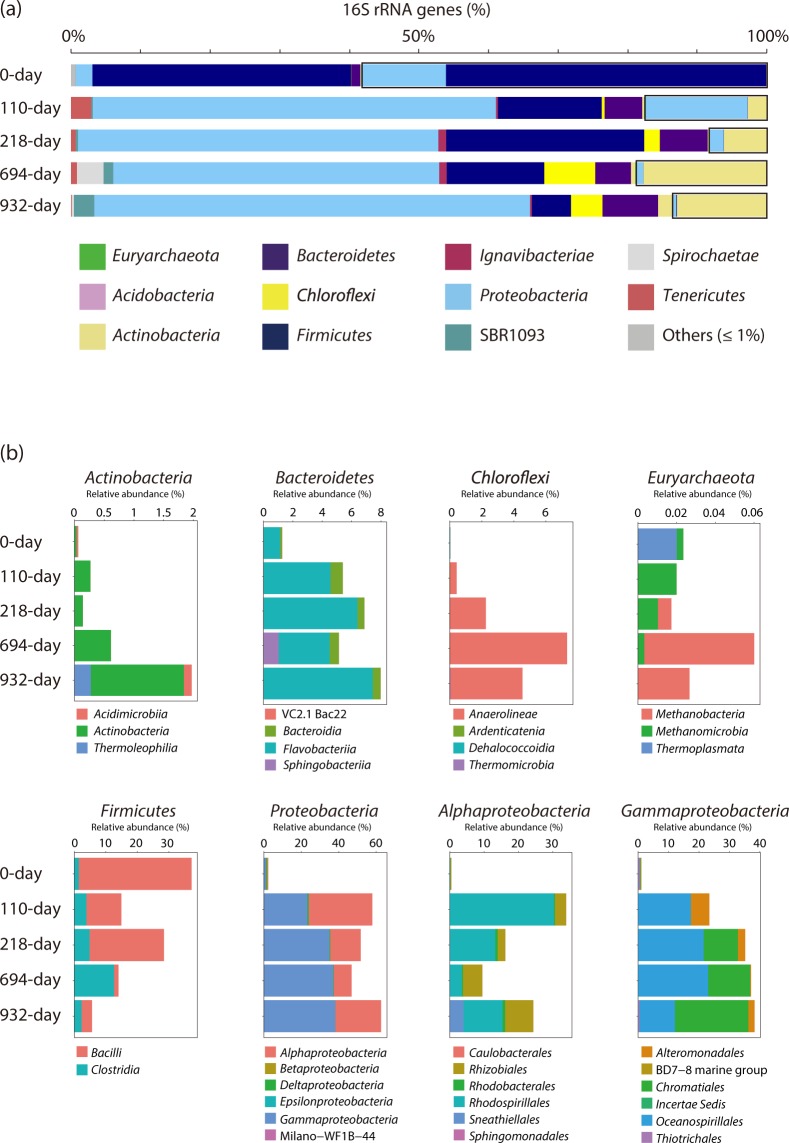
Microbial community structures in the DHS reactor based on SSU rRNA gene tag sequencing analyses. (**a**) Phylum-level taxonomic composition. Black-rimmed boxes indicate potential contaminant populations. (**b**) Class- or order-level taxonomic composition of the major bacterial groups and the phylum *Euryarchaeota*. The class- or order-level taxonomic compositions do not include potential contaminant populations. Relative abundance ratio was calculated using the total sequence read numbers including potential contaminant sequences. For *Gammaproteobacteria*, the orders *Aeromonadales*, *Cellvibrionales*, *Legionellales*, *Methylococcales*, *Pseudomonadales*, *Vibrionales*, and *Xanthomonadales* are not shown as their relative abundance ratios are quite low (≤0.06%).

**Figure 6:**
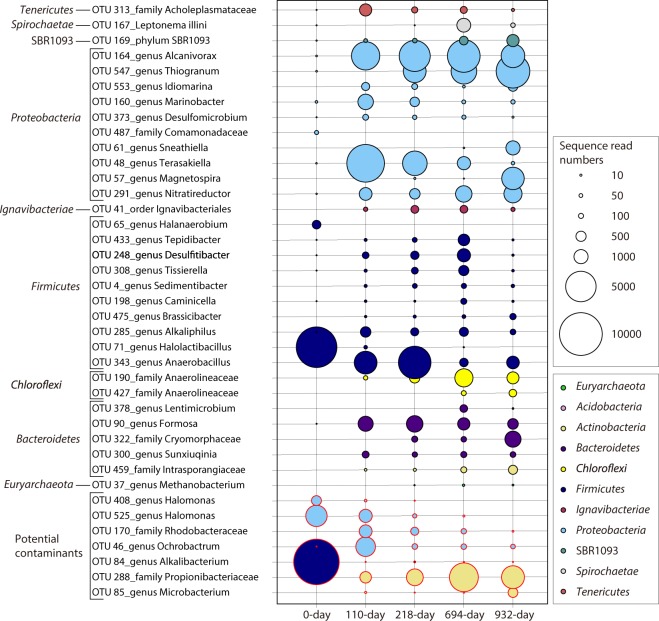
Shift of abundant OTUs throughout the reactor operation time. The size of each dot indicates the sequence read numbers. The taxonomic names before OTUs are the lowest-rank taxonomic group names as defined by the ARB Silva 128 database.

To monitor the microbial community composition during DHS bioreactor operation, small subunit (SSU) rRNA gene tag sequencing analysis was performed. Tag sequencing analysis results are summarized in Figs 5, 6, S3 and S4, and Supplementary Tables S4–S6.

Approximately 60% of the SSU rRNA gene sequences detected in the inoculum sample (i.e., 0-day sample in Fig. 5) was attributed to potential contaminant bacterial sequences (Fig. 5, Supplementary Tables S5 and S6). Major potential contaminant operational taxonomic units (OTUs) were *Alkalibacterium* of *Firmicutes* (OTU84) and *Halomonas* of *Gammaproteobacteria* (OTU525), accounting for 45.0% and 9.5%, respectively. Almost all potential contaminants were supposedly derived from the drilling mud and/or experimental procedures^23^. In total, 97 OTUs likely derived from indigenous deep subseafloor populations were detected in the inoculum sample (excluding singleton OTUs, Supplementary Table S4). Major OTUs (>1% in the 0 day inoculum library) belonged to the phyla *Proteobacteria*, *Firmicutes*, and *Bacteroidetes*. Archaeal sequences were also detected as minor components from the inoculum sample; e.g., candidate phylum Woesearchaeota (OTU78), an uncultured group of *Thermoplasmata* (OTU400), candidate phylum Lokiarchaeota (OTU538) and anaerobic methane-oxidizing archaea belonging to ANME-2d clade (OTU139).

During enrichment in the DHS reactor, the microbial community structure changed drastically (Figs 5 and 6, Supplementary Fig. S4, and Tables S5 and S6). Relative sequence abundance of the potential contaminant OTUs sharply decreased and accounted for only 8.3% to 18.8% of the population. However, several potential contaminants (e.g., OTUs 46, 85, and 288) became the predominant populations as cultivation continued (Fig. 6). During cultivation, we detected 149 OTUs of SSU rRNA gene sequences that were potentially derived from indigenous microbial populations (excluding singleton OTUs, Supplementary Table S5). Those within the phyla *Proteobacteria* (46.8–62.6% in the sponge carrier libraries), *Firmicutes* (5.6–28.4%) and *Bacteroidetes* (5.1–8.0%) dominated through the cultivation period, with OTUs belonging to the phyla *Actinobacteria* (0.1–2.0%), *Chloroflexi* (0.4–7.3%), *Ignavibacteriae* (0.3–1.1%), *Spirochaetes* (0–3.8%) and *Tenericutes* (0.1–2.9%) and the candidate phylum SBR1093 (0.2–2.9%) also becoming the predominant populations (>1% in any of the sponge carrier libraries) (Fig. 5, Supplementary Table S6). Especially, the following OTUs of potentially indigenous microorganisms were detected as major populations (>5% in any of the sponge carrier libraries) (Fig. 6, Supplementary Table S5): OTU164 (*Alcanivorax* of *Gammaproteobacteria*), OTU547 (*Thiogranum* of *Gammaproteobacteria*), OTU48 (*Terasakiella* of *Alphaproteobacteria*), OTU57 (*Magnetospira* of *Alphaproteobacteria*), OTU291 (*Nitratireductor* of *Alphaproteobacteria*), OTU343 (*Anaerobacillus* of *Firmicutes*), OTU190 (*Anaerolineaceae* of *Chloroflexi*), OTU90 (*Formosa* of *Bacteroidetes*), and OTU322 (*Cryomorphaceae* of *Bacteroidetes*). Certain major OTUs were closely related to obligatory aerobic or microaerophilic isolates (i.e., OTUs 547, 48, and 57). Although effluent media ORP values indicated the preservation of anaerobic conditions in the bioreactor column (Supplementary Fig. S1), molecular oxygen contamination cannot be completely ruled out over the long-term operation period. It is also possible that those aerobic microbes have anaerobic metabolisms.

Methanogen-related sequences were detected throughout the cultivation period, most of which were affiliated with a hydrogenotrophic methanogen of genus *Methanobacterium*. The *Methanobacterium* sequences were classified into two OTUs, although the great majority was affiliated with OTU37 (whereas OTU213 was detected only as single-sequence read in the 110-day effluent library). Relative sequence abundance of OTU37 was low in the sponge carrier libraries (<0.06%), but relatively high in the effluent libraries (0.3–9.0%), suggesting that *Methanobacterium* species actively proliferated in the reactor. Alternatively, *Methanbacterium* species grew in the liquid phase of sponge (=medium in pore space of the sponge carriers) without adhesion to the lignite particles because they used formate or hydrogen which are dissolved end products from lignite. In the pre-cultivation samples, no methanogen-related sequences were detected among the selected 30000 sequence reads (Supplementary Table S4). However, the original core sample of lignite 25R-2 yielded two identical sequences of OTU37 (data not shown). This result suggests that the predominant *Methanobacterium* in the DHS reactor originated from the 2-km-deep lignite layer. Additionally, the OTUs related with aceticlastic/methylotrophic methanogen *Methanosarcina* and aceticlastic methanogen *Methanosaeta* were detected in the 110-day sponge carrier and 932-day effluent libraries, respectively. Both methanogen-related sequences were minor components of the tag sequence libraries and they were retrieved as a single-read sequence (data not shown in Supplementary Table S5, because the sequences were not chosen among 30000 representative sequences from each library). Sequences related to the recently discovered methoxydotrophic methanogen^31^ were not detected in this study.

No OTU classified in the domain *Eukarya* was detected in the tag sequencing libraries. Moreover, no PCR products using 18S rRNA gene-targeted PCR primers were obtained from any of the reactor-enriched samples.

### Alpha diversity in the DHS reactor enrichments

To evaluate the diversity and richness of the DHS reactor enrichment samples, Chao 1 species richness, abundance-based coverage estimator (ACE), Shannon diversity index, Simpson’s evenness, tag sequence library coverage, and rarefaction curves were calculated for all the tag sequence libraries (Supplementary Tables S7 and Fig. S5). Additionally, we performed the same evaluation for zero-radius OTUs (ZOTUs) using the UNOISE algorithm^32^ (Supplementary Tables S7). Both conventional 97% cut-off OTU and ZOTU results showed similar trends regardless of potential contaminant sequence inclusion. Chao1 species richness and ACE scores decreased in the beginning of bioreactor cultivation (110 days of reactor operation), then increased, and became stable. Rarefaction curves exhibited similar behavior (Supplementary Fig. S5) whereas Shannon diversity index and Simpson’s evenness scores increased after cultivation. The alpha-diversity analyses suggest that some major microbial populations could not grow whereas minor microbial populations, comprising small or undetectable components in the inoculum samples, increased in the bioreactor.

### Isolation of anaerobic microorganisms from the DHS reactor

**Figure 7:**
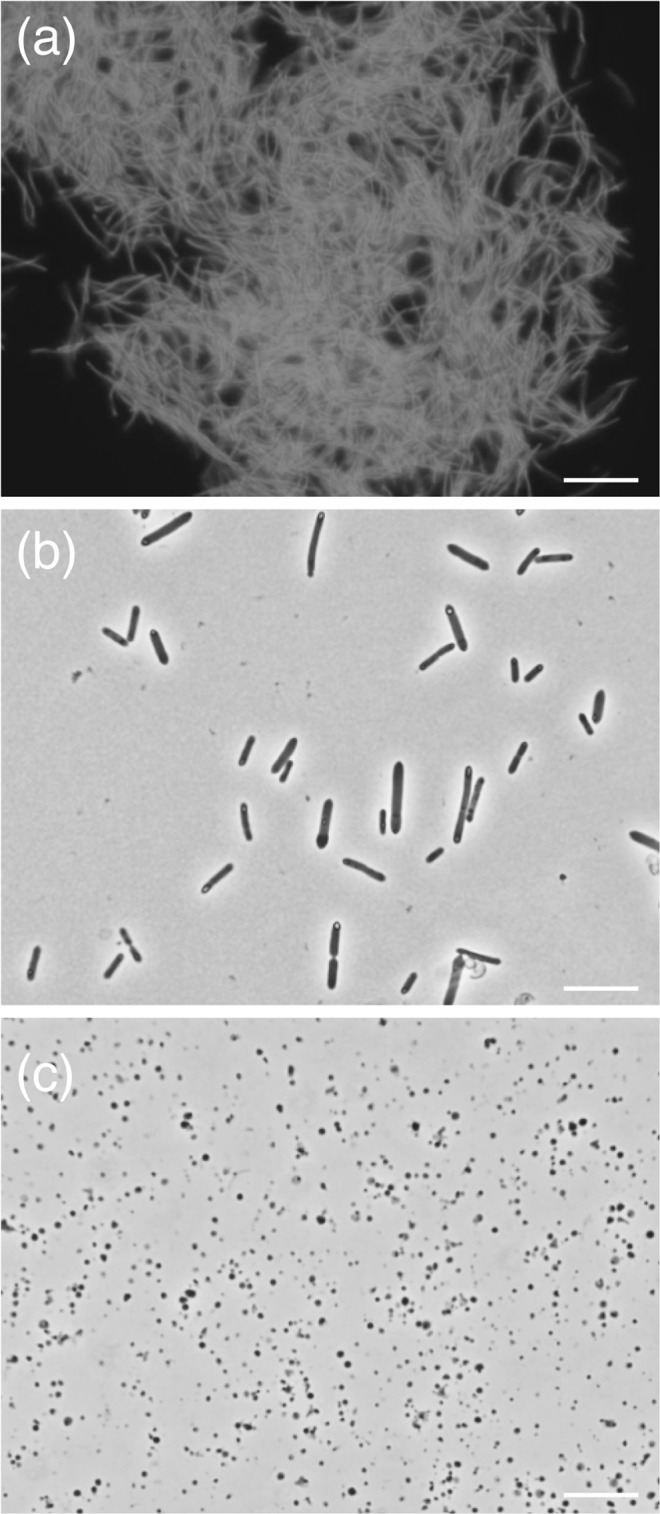
Photomicrographs of anaerobic microorganisms isolated in this study. (**a**) Fluorescence micrograph of *Methanobacterium* sp. strain MZ-A1, which grown on H_2_/CO_2_ medium at 40 °C. The methanogen cells produce autofluorescence derived from coenzyme F_420_. (**b**) Strain MZ-F1, belonging to the genus *Tepidibacter*, grown on yeast extract medium at 40 °C. (**c**) Strain MZ-XQ, belonging to the class *Mollicutes*, grown on glucose and yeast extract medium at 40 °C. Bars represent 10 µm.

To obtain pure cultures of anaerobic microorganisms from the DHS reactor, subsequent batch-type cultivations were performed using the enriched methanogenic community as inoculum (Supplementary Table S8). Cell growth occurred in H_2_-, formate-, and yeast extract-fed media within 2 weeks at 40 °C. Methane production was observed from the H_2_− and formate-fed cultures. Both methane-producing cultures contained rod-shaped, F_420_-autofluorescent methanogens that morphologically resembled *Methanobacterium* as the predominant population. The yeast extract-fed culture contained several morphologically distinct microbial cells. After three successive transfers, we performed serial-dilutions in both liquid and solid media for microorganism isolation, resulting in a methanogen isolate from the H_2_-fed culture and two anaerobic bacteria from the yeast extract-fed culture (Fig. 7, Supplementary Table S8). The isolated methanogen is closely related to *Methanobacterium* sp. strain MO-MB1 (16S rRNA gene sequence identity of 100%), which was isolated from shallow sediments at Site C0020^19^. One of the two anaerobic bacteria isolated from the yeast extract-fed culture is closely related to *Tepidibacter mesophilus* within the phylum *Firmicutes* (99.4% 16S rRNA gene sequence identity); the other possibly represents a new species of the class *Mollicutes* within the phylum *Tenericutes*. The closest cultured representatives are strain MO-XQ, also isolated from shallow sediments at Site C0020^19^ (99.4% 16S rRNA gene sequence identity) and *Acholeplasma palmae* (92.6%). Sequences identical to those of the isolate were detected in the bioreactor enrichment (OTUs 37, 433, and 313). No clear cell growth or substrate consumption was observed from other cultures after 6 months of incubations. Moreover, we set up cultures incubated at higher temperatures (55 °C and 80 °C) to address whether the enriched methanogenic community contains thermophilic microorganisms or not. However, no cell growth was observed after 2 months of incubation.

## Discussion

In this study, we successfully obtained a methanogenic microbial community from 2-km-deep subseafloor coalbed layer samples. Increased sequence frequency of some indigenous populations that were present in the inoculum samples (Figs 5 and 6, Supplementary Table S5) clearly indicated maintenance of cellular growth and population in the DHS reactor, suggesting that the continuous-flow DHS reactor system could be a powerful tool to cultivate indigenous microbial communities from the deep subseafloor sedimentary biosphere. The unique feature of the polyurethane sponge carrier contributed to the successful cultivation of a microbial community from lignite samples. Based on a previous study using subseafloor sediment^21^, we first assumed that the microorganisms enriched from the lignite samples would grow on the sponge surface and within its pore space. However, as shown in Fig. 4 and Supplementary Fig. S2, the microbes colonized entirely on the lignite particle surfaces. The polyurethane sponge carriers functioned to retain lignite particles in the reactor column for long-term continuous-flow cultivation, whereas the lignite particles themselves served as microbial habitats. Consequently, the DHS reactor system is also applicable to cultivate fastidious microorganisms on solid substances such as coals, rocks, and minerals.

Together, our chemical data is consistent with the existence of a microbial ecosystem that degrades lignite-derived organic material to diverse intermediate organic substances (e.g., acetate and high molecular weight, humic-like aromatic compounds) and methane as metabolic end products (Figs 2 and 3, Supplementary Table S1). For example, the remarkable increase in methane concentration at around 600–700 days of the reactor operation shows a clear link between chemical and microbial data (Fig. 1a). The δ^13^C values of the methane decreased sharply during this period (Fig. 2b), indicating the occurrence of active CO_2_ reducing methanogenesis. Shift of effluent pH values from approximately 7.3 to 7.8 (Fig. S1b) and an increase of the detection frequency of *Methanobacterium* sequences in the SSU rRNA gene-tag sequencing libraries (Fig. 5b) also support the presence of active CO_2_ reducing methanogenesis during this time. This active methanogenesis should be supported by the supply of methanogenic substrates (i.e., hydrogen and formate) that would be generated by active lignite-degradation. Microbial lignite-degradation in the reactor indeed was demonstrated by the increase of the AC/M ratio (Fig. 3c).

We interpret acetate to be a fermentation product of lignite-derived organic matters as acetate δ^13^C values were similar to those of higher-plant derived saturated hydrocarbons detected within natural lignite samples of Site C0020^33^. No known homoacetogen-related sequences in the SSU rRNA gene-tag sequence libraries (Supplementary Table S5) and absence of homoacetogen growth in the batch-type cultivation using the reactor enrichments (Supplementary Table S8) also suggests that acetate is primarily produced via fermentation rather than by homoacetogenesis^25^. EEM fluorescence spectroscopy analysis also indicated humic-like compounds as precursors for fermentation and that an ongoing turnover of these compounds is driven by microbial activity (Fig. 3).

The majority of the methane produced in the DHS reactor is most likely generated via hydrogenotrophic (CO_2_ reduction) methanogenesis. Carbon isotope fractionation between lignite-derived methane (minimum δ^13^C of −94.0‰; mean of −68.1‰) and organic matter (δ^13^C of −28‰ to −36.5‰)^33^ was approximately about 70‰ at maximum, which is equivalent to that between methane and CO_2_ in hydrogenotrophic methanogenesis under mesophilic, H_2_-limited conditions^34^. However, the δD values of the methane (−232.1 to -393.0‰) point to the potential contribution of aceticlastic/methylotrophic methanogenesis^24,35,36^. The methylotrophic signals are consistent with recent findings of position-specific ^13^C enrichments of methoxy groups within the Shimokita lignites from Site C0020^37^, consistent with their microbial utilization by methanogens. Moreover, as the δ^13^C values of methane fluctuated and sometimes became isotopically heavy (from 210 to 455 days, from 693 to 721 days, and after 805 days, Fig. 1b), aceticlastic/methylotrophic methanogenesis likely co-occurs next to hydrogenotrophic methanogenesis resulting in smaller stable carbon isotope fractionation^24^. The activity of both pathways was supported by SSU rRNA gene tag sequencing analysis, in which minor but archaeal OTUs were affiliated with aceticlastic/methylotrophic methanogen *Methanosarcina* and aceticlastic methanogen *Methanosaeta* together with the predominant OTUs of hydrogenenotrophic methanogen *Methanobacterium*.

The enriched microbial community comprised phylogenetically diverse microorganisms (Figs 5, 6 and Supplementary Fig. S3, and Tables S4–S6). During the cultivation period, major OTUs (>1% in any of the sponge carrier libraries) were affiliated with the phyla *Proteobacteria*, *Firmicutes*, *Chloroflexi*, *Actinobacteria*, *Bacteroidetes*, *Spirochaetes*, *Tenericutes*, *Ignavibacteriae*, and SBR1093. The phylum-level community composition was similar to those reported in the previous molecular studies of methanogenic communities in coal environments^38–42^ and enriched communities from coal and its associated water^43–47^. However, the detailed composition of microbial community members enriched in our reactor at below the phylum level differed from the previously reported compositions, potentially due to the different sources of the inoculum and enrichment conditions (e.g., marine vs. terrestrial).

When comparing detection frequency of the dominant OTUs in the sponge carrier and effluent samples, several OTUs were exclusively detected from either the sponge carrier or effluent samples (Supplementary Table S5). This result suggests that two types of microbial communities exist in the DHS reactor: one is the sessile community with members attached to the lignite surface and, depending on the substrates, inhered in lignite; the other one is the planktonic community with members relying on the substrates released from the degradation of lignite. This observation also suggests that stepwise biodegradation of organic substances in lignite were carried out in the DHS reactor, but inferring the detailed metabolic functions of each host microorganisms for the OTUs is difficult only from the SSU rRNA gene tag sequencing data (Supplementary Table S5). Therefore, to address this, further polyphasic investigation (e.g., subsequent isolation, metagenome/transcriptome analyses and stable isotope labeling experiments) using the DHS reactor enrichment culture is needed. Although the metabolic functions are not yet clarified for each microbial species at this moment, the host microorganisms for most of the predominant OTU probably play an important role in the degradation of coal-derived organic substances. For example, OTUs of the class *Clostridia* were frequently detected from the reactor samples at different times (e.g., OTUs 433, 285 and 198, Fig. 6 and Supplementary Tables S5). In general, *Clostridia* members are strictly anaerobic bacteria that degrade a wide variety of heterotrophic organic compounds^48,49^; therefore, the reactor-enriched *Clostridia* bacteria may be relevant to the lignite-degradation. A *Clostridia* strain capable of decomposing polycatechol and humic substances was isolated from groundwater samples, including groundwater associated with a coal bed recovery site^50^. Likewise, other predominant OTUs belonging to *Gammaproteobacteria*, *Alphaproteobacteria*, *Actionobacteria*, and *Bacteroidetes* may be associated with the degradation of lignite-derived organic matter^40,51^, as the bacterial groups contain metabolically versatile bacteria. Notably, two predominant OTUs are closely related to hydrocarbon-degrading bacterial groups: n-alkanes degrader *Alcanivorax* (OTU164) and polycyclic aromatic hydrocarbon degrader *Nitratireductor* (OTU291). Those genera are generally recognized as aerobic microorganisms, but some strains can grow under anaerobic conditions^52–54^. Oxygen contamination could not be excluded from long-term reactor operation. Therefore, the presence of microbes degrading hydrocarbons using contaminant oxygen cannot be completely ruled out. On the other hand, a previous metagenomic study has identified high gene proportions for aerobic hydrocarbon metabolism enzymes in Canadian subsurface coalbed samples^55^. Therefore, there is a possibility that those microorganisms perform hydrocarbon-degradation under anaerobic conditions.

We successfully obtained three isolates including a methanogen from the DHS reactor enrichment (Fig. 7). Identical 16S rRNA gene sequences with indigenous population-derived OTUs 37, 313, and 433 (Fig. 6, Supplementary Table S5) support that these isolates originated from the 2-km-deep coalbed layers. Notably, the reactor isolates, *Methanobacterium* sp. strain MZ-A1 and *Tenericutes* sp. strain MZ-XQ share (almost) identical 16S rRNA gene sequences with *Methanobacterium* sp. strain MO-MB1 (100% sequence identity) and *Tenericutes* sp. strain MO-XQ (99.4%), respectively, both of which were isolated from shallow subseafloor sediments of the same drilling site in our previous study^19^. To examine if the evolutionary genome diversification is potentially affected by the geological time (approximately 20 million years of difference is estimated between the deposition ages of 2-km-deep coalbed layer and shallow subseafloor sediments), we are currently performing comparative genome analysis and physiological characterization for both “deep isolates” (i.e., strains MZ-A1 and MZ-XQ) and “shallow isolates” (i.e., strains MO-MB1 and MO-XQ).

It is possible that deep subseafloor coalbed environments harbor more extant and diverse microorganisms than estimated herein. We performed alpha-diversity analysis of SSU rRNA tag sequencing data to estimate cultured microbial community diversity and richness in the DHS reactor (Supplementary Table S7). Notably, Shannon diversity index and Simpson’s evenness scores increased along with the reactor operation time, although enrichment cultivation generally exhibits a strong selective bias for microbial populations that can adapt to cultivation conditions. Conversely, species richness scores (i.e., Chao 1 and ACE) decreased immediately upon initial bioreactor cultivation, then increased and became stable. Generally, species richness scores decrease as enrichment cultivation proceeds. These results suggest that some predominant microbial populations in the inoculum sample could not grow, but many minor microbial populations could grow in the bioreactor. Specifically, we identified 97 and 149 OTUs, apparently derived from indigenous populations, from the samples before and after cultivation, respectively (excluding singleton OTUs, Supplementary Tables S4 and S5). Thus, microbial diversity in the inoculum samples was estimated to be low, although various indigenous populations were originally present in the inoculum samples. The low diversity estimation in the inoculum sample might be derived from (i) insufficient amount of DNA for PCR amplification obtained from most microorganisms owing to low abundance^23^; (ii) insufficient sequence read numbers to cover the entire microbial community in the inoculum samples; and/or (iii) possible DNA extraction and PCR amplification biases affecting the tag sequencing results.

Specifically, DNA extraction bias may have a significant impact on the estimation of microbial diversity, specifically in the inoculum sample. DNA extraction from deeply buried microbial cells is known to be difficult as compared to from other habitats, possibly because of rigid cell forms^56^. Using a standard DNA extraction kit, Morono *et al*.^57^ demonstrated that at least 70% of subseafloor sedimentary microbial cells remained intact in DNA extraction residue. This suggests that many subseafloor sedimentary microbes possess a rigid cell envelope to provide resistance against common DNA extraction chemicals. Moreover, Lomstein *et al*.^58^ reported abundance of bacterial endospores in deep subseafloor sediments. Endospores are dormant and mechanically tough structures produced by certain members of bacteria within phylum *Firmicutes*^59^. We also detected members of the phylum *Firmicutes* that were closely related to endospore-forming members in the inoculum and enriched samples (Supplementary Tables S5). Endospores are unlikely to be detected by either nucleic acid fluorescence staining^60,61^ or rRNA-targeted fluorescence *in situ* hybridization techniques^62,63^. They are also resistant to physical-chemical cell lysis procedures of common DNA extraction methods^64^. Therefore, microbial components from endospores and rigid cell envelope forms may withstand DNA extraction and result in the underestimation of microbial diversity estimation in the pre-cultivation samples (Supplementary Tables S7). However, in the continuous-flow bioreactor, microbial cell membrane permeability and fluidity likely increased to incorporate energy substrates for active growth, potentially enhancing DNA yield, Shannon diversity index, and Simpson’s evenness scores in the enriched samples (i.e., Supplementary Tables S2 and S7). Thus, performing molecular-based analyses of microbial diversity not only on natural samples, but also on cultivated or enriched samples under the similar conditions to their natural habitat would provide important clues to clarify the microbial diversity in the deep subseafloor sediments.

In summary, our >1000-day-long DHS bioreactor operation for 2-km-deep, 20-million-year-old lignite core sample demonstrates that a good fraction of the deeply buried subseafloor microbial community is cultivable, including key players in the anaerobic heterotrophic microbial ecosystem, such as various fermenters and methanogens. The enriched community consists of phylogenetically diverse microorganisms and possibly contains a concert of microbes that can convert complex coaly organic matters to methane. The enriched microbial community may be applicable to biological techniques that stimulate methane-production from low-rank coals and coalbed methane layers^40,65–69^. The cultured methanogenic community thus represents an attractive microbial entity for both science and engineering.

## Methods

### Sediment core sample

In 2012, the IODP Expedition 337 “Deep Coalbed Biosphere off Shimokita” was conducted at approximately 80 km offshore the Shimokita Peninsula, Japan, in the northwestern Pacific using the deep-sea drilling vessel *Chikyu* at Site C0020 (41°10.5983′N, 142°12.0328′E, 1180 m water depth). The detailed site information and coring operation have been described previously^23,33,70–73^. In the present study, we used three whole-round core samples obtained from approximately 2000 mbsf; i.e., two lignite core samples (1922 and 1998 mbsf) and a sandstone core sample (1978 mbsf). The temperature at 2 km below seafloor *in situ* was about 48 °C^71,74^. Samples used for inoculation were obtained from the innermost section of the whole-round core using sterilized ceramic knives and tip-cut plastic syringes in a laminar flow clean bench onboard the *Chikyu*. The sandstone and lignite samples were preserved in sterile glass bottles under anaerobic conditions with filtered N_2_ gas, and stored at 4 °C in the dark prior to use in the shore-based laboratory.

### DHS bioreactor and operation

A schematic diagram of the DHS reactor is shown in Fig. 1. The previous DHS reactor columns were made of polyvinyl chloride^19,20^; in this study, however, we used a glass column (diameter 12 cm; length 50 cm) to maintain strict anaerobic conditions. For the joint parts of tubing lines from the medium storage bottle to the top of the reactor column, we used metal tube-fittings and tube-adapters (Swagelok, Solon, OH, USA) to prevent potential oxygen contamination. Polyurethane sponge-cubes (2 cm × 2 cm × 2 cm, pore size 0.83 mm) were used as the carrier material for creating microbial habitats. The sponge cubes were encased in plastic nets to prevent crushing of the sponges. A total of 100 sponge carriers were randomly packed into the glass column. The sponge carriers were not submerged and freely placed in the atmosphere. The total pore volume of the sponge was 800 ml; this volume was used for calculating the HRT.

A mixture of the crushed lignite and sandstone samples was used for the reactor cultivation. To prepare the master slurry, the surface of core samples was peeled again using sterilized ceramic knives, and then crushed and pulverized with sterilized hammers and tungsten carbide lined mortars (Nichika Inc., Kyoto, Japan). Three samples were mixed (50 g of 1922 mbsf lignite, 10 g of 1978 mbsf sandstone, and 50 g of 1978 mbsf lignite) with 890 ml anaerobic medium (described below) without any organic substances. All of the inoculum sample preparation was performed in an anaerobic chamber (Coy Laboratory Products, Grass Lake, MI, USA). The sponge carriers were soaked with the master slurry sample manually and placed into the glass column in a cold room maintained at 4 °C. The master slurry and glass column were continuously flushed by N_2_ gas at all times. After inoculation, the glass column was tightly closed and installed in an incubator (LTI-1200E, EYELA, Tokyo, Japan) in the dark at 40 °C.

The composition of the medium for the DHS reactor was as follows (l^−1^): 5.9 mg sodium acetate, 7.3 mg sodium propionate, 8.7 mg sodium butyrate, 10 mg yeast extract, 0.53 g NH_4_Cl, 0.1 g KH_2_PO_4_, 4 g MgCl_2_·6H_2_O, 1 g CaCl_2_·2H_2_O, 20 g NaCl, 2 g NaHCO_3_, 0.1 g Na_2_S·9H_2_O, 2 ml Ti(III)-nitrilotriacetate, 1 ml trace element solution^75^, 1 ml vitamin solution^76^, and 1 ml resazurin solution (1 mg ml^−1^). The detailed reasons for providing these organic substances for the initial phase of the DHS reactor operation are provided in Supplementary Text 1. The total concentration of dissolved organic matter in the medium was 1.13 mM-C. After 694 days of the reactor operation, we stopped adding organic regents (i.e., acetate, propionate, butyrate, and yeast extract) to the medium. The medium was purged by N_2_ gas and pH was adjusted to 7.5. Five liters of medium was made once a week. The 5 l medium bottle was stored at 10 °C in the dark. Detailed reactor operation is described in the Supplementary Text 2.

### Chemical analysis and Sampling from the DHS reactor

Detailed chemical analysis method is described in the Supplementary Text 2, except for EEM spectroscopy analysis^75,76^.

For EEM spectroscopy analysis, all effluent seawater samples were filtered with a 0.22 µm pore-size polyethersulfone filter unit (Millipore, Billerica, MA, USA) immediately after sampling and stored at 4 °C or −20 °C until measurements. Fluorescence measurements were performed on an Agilent Cary Eclipse fluorescence spectrophotometer (Agilent Technologies, Santa Clara, CA, USA) at room temperature in a 1 cm quartz fluorescence cell. The integral area of the Raman peak (excitation 350 nm) was calculated using Milli-Q water as a reference. A range of emission spectra spanning 300–530 nm was recorded while exciting at wavelengths in the range 230–410 nm. EEMs of 67 samples were modeled by parallel factor analysis (PARAFAC) using MATLAB software^77^. The appropriate number of components was validated by half-value analysis. Five individual fluorescent components have been identified in our study combined with PARAFAC^78^: protein-like peaks (P) and humic-like peaks M, C and A (together with a side peak of C).

The fluorescence index is the ratio of the fluorescence intensity at 450 to 500 nm emission excited at 370 nm. Terrestrial-derived humic-like compounds like plant-lignin derived phenols are more conjugated and emit longer fluorescence emission wavelengths (em = ~450 nm) than microorganism-derived humic compounds (em = ~400 nm). Consequently, FI of less than 1.4 indicates microbially derived DOM while FI > 1.8 indicates DOM derived from higher plant material^79^. The biological index is the ratio of fluorescence intensity emitted at emission 380 nm and the maximum of intensity at emission 430 nm exited at 310 nm, a biological index > 1 indicates fresh autochthonous (microbial) DOM production^80^. The humification index (HIX) is calculated from the ratio of integrated fluorescence emission in 435–480 nm to that in 300–345 nm, indicating humified DOM with HIX more than 10 and autochthonous DOM with HIX less than 4^80^.

Sponge carriers and effluent were sampled at 37, 110, 218, 694 and 932 days of operation (the 37-day sample was only for effluent). At each sampling of sponge carriers, the lid of the reactor column was opened and five sponge carriers were randomly collected using a sterilized tweezers. During the sampling of sponge carriers, N_2_ gas was flushed through the bioreactor column. After sampling, the sponge carriers were squeezed by a sterilized tweezers to collect lignite particles attached to the sponge carriers and media retained in the sponge carriers. These collected lignite particles and media were mixed and used in the microbial community analyses as sponge carrier samples.

### Microscopy

Microbial cell morphology was examined under a fluorescence microscope (Olympus BX51F, Tokyo, Japan) equipped with a color CCD camera system (Olympus DP72). For scanning electron microscopic (SEM) images, lignite particle and sponge samples were prefixed in 2.5% (w/v) glutaraldehyde in the anaerobic medium used for the DHS reactor cultivation without organic compounds. After the samples were rinsed with distilled water, the conductive staining was performed by incubation in 0.2% aqueous tannic acid (pH 6.8) for 30 min, the samples were then washed with distilled water and treated with 1% aqueous osmium tetraoxide for 1 hour. Subsequently, the samples were dehydrated in a graded ethanol series and critical point dried in a JEOL JCPD-5 (Tokyo, Japan). The samples were then coated with osmium using an osmium plasma coater (POC-3; MEIWAFOSIS Co., Ltd., Tokyo, Japan) and observed with a JEOL JSM-6700F field emission SEM operated at 5 kV.

### DNA extraction, PCR amplification, and SSU rRNA gene tag sequencing analysis

DNA extraction and PCR mixture preparation were performed under a KOACH T 500-F tabletop air filtration system with a static remover (KOKEN Ltd., Tokyo, Japan) to reduce contamination. To extract DNA from the samples, an ISOL for Beads Beating kit (Nippon Gene, Tokyo, Japan) was used according to the manufacturer’s protocol. The concentration of extracted DNA was measured using a Quant-iT dsDNA High-Sensitivity Assay Kit (Life Technologies, Carlsbad, CA, USA). PCR amplification was performed using TaKaRa LA *Taq* (TaKaRa Bio Inc., Kusatsu, Shiga, Japan), and the reaction mixtures for PCR were prepared according to the manufacturer’s instruction. For PCR amplification, a universal primer pair 530 F/907R^81^ was used to amplify SSU rRNA genes (V4-V5 regions). The PCR primers contained overhang adapters at the 5′ ends. PCR amplification conditions were as described previously^82^. Detailed following procedures for sequencing and data analysis as described Supplementary Text 2.

### PCR amplification of 18S rRNA gene

PCR amplification of 18S rRNA gene for bioreactor enrichment samples is also described in Supplementary Text 2.

### Isolation of anaerobic microorganisms from the DHS bioreactor

Detailed isolation procedure of anaerobic microorganisms is described in the Supplementary Text 2. The isolates obtained in this study have been deposited in the Japan Collection of Microorganisms (JCM 32063 for *Methanobacterium* sp. strain MZ-A1; JCM 32064 for *Tepidibacter* sp. strain MZ-F1; JCM 31639 for *Tenericutes* sp. strain MZ-XQ).

### Nucleotide sequence accession numbers

The SSU rRNA gene tag sequence data reported in this manuscript was deposited in Bioproject PRJNA355906 with the accession numbers SPR5091819–SPR5091842. The 16S rRNA gene sequences of isolates obtained in this study have been deposited in the DDBJ/EMBL/GenBank database under accession numbers LC214858-LC214860.

## Supplementary information

Supplementary Texts and Figures

Supplementary Tables
