# Supplementary material for: Cultivable microbial community in 2-km-deep, 20-million-year-old subseafloor coalbeds through ~1000 days anaerobic bioreactor cultivation
Source: Sci Rep. 2019 Feb 19;9:2305. doi: 10.1038/s41598-019-38754-w (PMC6381156; doi:10.1038/s41598-019-38754-w)
Supplement: Supplementary file 1 — Supplementary Texts and Figures [file 41598_2019_38754_MOESM1_ESM.pdf]

Supplementary Information

Cultivable microbial community in 2-km-deep, 20-million-year-old subseafloor coalbeds through ~1000 days anaerobic bioreactor cultivation

Hiroyuki Imachi[1,2*], Eiji Tasumi[1], Yoshihiro Takaki[1,3], Tatsuhiko Hoshino[2,4], Florence Schubotz[5], Shuchai Gan[5], Tzu-Hsuan Tu[1,6], Yumi Saito[1], Yuko Yamanaka[1], Akira Ijiri[2,3], Yohei Matsui[2,3], Masayuki Miyazaki[1], Yuki Morono[2,4], Ken Takai[1,2], Kai-Uwe Hinrichs[5], and Fumio Inagaki[2,4,7]

[1] Department of Subsurface Geobiological Analysis and Research (D-SUGAR), Japan Agency for Marine-Earth Science and Technology (JAMSTEC), Yokosuka, Kanagawa 237-0061, Japan.

[2] Research and Development Center for Submarine Resources, JAMSTEC, Yokosuka, Kanagawa 237-0061, Japan.

[3] Project Team for Development of New-generation Research Protocol for Submarine Resources, JAMSTEC, Yokosuka, Kanagawa 237-0061, Japan.

[4] Kochi Institute for Core Sample Research, JAMSTEC, Nankoku, Kochi 783-8502, Japan.

[5] MARUM Center for Marine Environmental Sciences and Department of Geosciences, University of Bremen, D-28359 Bremen, Germany.

[6] Institute of Oceanography, National Taiwan University, Taipei 106, Taiwan.

[7] Research and Development Center for Ocean Drilling Science, JAMSTEC, Yokohama, Kanagawa 236-0001, Japan.

*Corresponding author: Hiroyuki Imachi

Supplementary Texts 1 and 2, Figures S1-S5.

Supplementary Text 1

Reasons for providing organic substances to the DHS reactor. In addition to lignite particles, we also provided acetate, propionate, butyrate, and yeast extract as potential energy and carbon sources for the enrichment in the DHS reactor. Acetate constituents an energy/carbon source for aceticlastic methanogenens and it is also an important carbon source for many hydrogenotrophic methanogens[83]. Yeast extract functions as a growth requirement for many methanogens and anaerobic heterotrophs[83]. Propionate and butyrate are expected to be intermediates of methanogenic biodegradation from coal, which is a complex organic compound[84]. Therefore, if propionate- and butyrate-degradations occur in the bioreactor, degradations of high molecule organic compounds derived from coal, such as aromatics, long chain alkanes, and long chain fatty acids, may be enhanced in terms of thermodynamics for the entire series of biodegradation reactions from coal to methane. Furthermore, in many natural anaerobic environments, including the subseafloor coal bed layers, hydrogenotrophic methanogens should thrive by receiving the H_2 that is provided by heterotrophic H_2-producing microorganisms, which catalyze the oxidation of a variety of organic substances[85,86]. The methanogens utilize the H_2 produced by these heterotrophic bacteria; in return, the bacteria benefit from the removal of excess H_2 that would otherwise inhibit their growth. This relationship is commonly referred to as interspecies H_2 transfer[86,87], with such syntrophic relationships between methanogens and heterotrophs likely existing in the subseafloor environment. Therefore, we assumed that the organic substances used in this study might act to increase metabolic activities of heterotrophic microorganisms and methanogens, which live in subseafloor coal bed environments.

References

83. Whitman, W.B. Bergey's Manual of Systematics of *Archaea* and *Bacteria*. Wiley Online Library. doi: 10.1002/9781118960608 (2015).

84. Jones, E. J. P., Voytek, M.A., Corum M.D. & Orem W.H. Stimulation of methane generation from nonproductive coal by addition of nutrients or a microbial consortium. *Appl. Environ. Microbiol.* 7**6**, 7013-7022 (2010).

85. Liu, Y, & Whitman, W.B. Metabolic, phylogenetic, and ecological diversity of the methanogenic *Archaea*. *Ann. N. Y. Acad. Sci.* **1125**, 171-189 (2008).

86. Stams, A.J.M. & Plugge, C.M. Electron transfer in syntrophic communities of anaerobic bacteria and archaea. *Nat. Rev. Microbiol.* **7**, 568-577 (2009).

87. Schink, B. Energetics of syntrophic cooperation in methanogenic degradation. *Microbiol.*

Mol. Biol. Rev. **61**, 262-280 (1997).

Supplementary Text 2

DHS bioreactor and operation. The medium was supplied into the reactor from the top inlet port using a peristaltic pump (Masterflex L/S tubing pump 7550-50, Cole-Parmer, Vernon Hills, IL, USA) with viton tubing (Cole-Parmer) and distributed to the sponge carriers by a distributor unit. The medium then flowed down, passing through the sponge carriers by gravity, and was finally pumped out of the glass column. A certain amount of the medium accumulated at the base of the bioreactor column prior to being drained out of the DHS reactor. The HRT in the reactor was set at 70 h and the medium was supplied intermittently by an automatic on/off timer (FT-011, Fine Inc., Tokyo, Japan, 1 min/9 min [on/off]) connected to the peristaltic pump to spread the medium to the sponge carriers throughout the glass column. It is noted that the time for the contact between medium fluid and sponge carrier/lignite might be shorter than 70 h because a previous study showed that actual HRT was shorter than theoretical one[88]. The distributor unit was manually rotated at three- or four-day intervals to diffuse the medium into all of the sponge carriers. The gas phase in the reactor was sampled from the top portion of the reactor. The DHS reactor was operated under atmospheric pressure.

Chemical analysis. The temperature, pH and oxidation-reduction potential (ORP) of effluent seawater were measured using an InPro3250 pH and redox sensor (Mettler Toledo, Columbus, OH, USA). Methane concentration in the bioreactor headspace was monitored and quantified using a gas chromatograph (GC-4000, GL Science Inc., Tokyo, Japan) equipped with a Shincarbon ST 50/80 column (1.0 m × 3.0 mm ID, Shinwa Chem. Ind., Kyoto, Japan) and a flame ionization detector with N_2 as a carrier gas. The stable carbon and hydrogen isotope compositions of methane in the sampled gas phase were analyzed as previously described30. Concentrations of dissolved organic carbon (DOC) were measured using a TOC analyzer (TOC-V WS, Shimadzu, Kyoto Japan) according to manufacturer's instruction. Concentrations of acetate, propionate, butyrate, and formate in effluent samples were measured by using a long- and short-chain fatty acid labeling kit (YMC Co., Kyoto, Japan) and a high performance liquid chromatography system consisted of an SCL-10A VP system controller, two LC-10Ai pumps, an SIL-20A autosampler, a CTO-10A VP column oven, and an SPD-10A VP UV-VIS detector (Shimadzu). The measurement conditions were as follows: column, Nova-Pak® C18 column (4 µm, 3.9 mm × 150 mm, Waters, Milford, MA, USA);

mobile phase, (A) water, (B) 0.1 vol% trifluoroacetic acid – acetonitrile; flow rate, 1 ml/min; gradient program, 2% B for 25 min, 2-9% B for 5 min, 9% B for 65 min, 9-100% B for 5 min, 100% B for 10 min, 100-2% B for 5 min, and 2% B for 5 min; detection, UV 400 nm; column oven temperature, 45ºC. The stable carbon isotope composition of acetate was determined by means of isotope-ratio-monitoring liquid chromatography mass spectrometry (irm-LCMS), using a Thermo-Finnigan Delta Plus XP isotope-ratio mass spectrometer connected to LC IsoLink, as described by Ijiri *et al.* (2012)[89].

DNA extraction, PCR amplification, and SSU rRNA gene tag sequencing analysis. After PCR amplification, PCR products were checked for size by electrophoresis on a 1.5% agarose gel using RedSafe stain (FroggaBio Inc., Toronto, Canada), purified using the ExoSAP-IT PCR Product Cleanup reagent (Affymetrix Inc., Cleveland, OH, USA), and quantified using a Quant-iT dsDNA High-Sensitivity Assay Kit (Life Technologies, Carlsbad, CA, USA). Then, a second PCR amplification was performed to add multiplexing indices and Illumina sequencing adapters. Ex Taq polymerase (TaKaRa Bio Inc., Kusatsu, Shiga, Japan) was used for the second PCR amplification. The adapter-attached PCR products were purified with Agencourt AMPure XP (Beckman Coulter, Brea, CA, USA) and quantified using a Quant-iT dsDNA High-Sensitivity Assay Kit (Life Technologies). Quality and DNA concentration of the PCR products were checked using a Bioanalyzer 2100 (Agilent Technologies, Santa Clara, CA, USA) and real-time PCR using a KAPA Library Quantification Kit (Kapa Biosystems, Wilmington, MA, USA). The PCR products were subjected to DNA denaturing and sample loading on a sequencer using MiSeq v3 reagent (Illumina, San Diego, CA, USA) according to the manufacture's protocol. Sequencing was performed using a MiSeq system (Illumina).

Paired-end reads were merged using PEAR[90]. PCR primers were removed from the merged sequences using Cutadapt v1.10. Low-quality (Q score <30 in over 3% of sequences) and short (< 150 bp) reads were filtered out using a custom perl script. SSU rRNA gene amplicon analysis from 30000 sequences sampled randomly was performed using the QIIME software package[91]. After the removal of chimeric sequences using Usearch61[92] in QIIME, OTUs were selected at the 97% similarity level using UCLUST[92] and were subsequently assigned to a taxon (at phylum, class, order, family, and genus levels) by comparison with the non-redundant 16S rRNA small subunit SILVA128 database. Alpha diversity indices were estimated using QIIME and non-metric multidimensional scaling (NMDS) analysis was performed based on the Bray-Curtis distance matrix using the function "metaMDS" from the vegan package in R (http://vegan.r-forge.r-project.org). Rarefaction curves were constructed

using the function "rrarefy" from the vegan package in R. In addition to analysis using conventional 97% cut-off OTUs, we also estimated alpha diversity indices using zero-radius OTUs obtained with the UNOISE2 algorithm[32]. Toward this end, the paired-end reads were quality-trimmed with AfterQC[93]. After the trimmed paired-end sequences were merged and screened by length, primer sequences were removed by using Mothur v.1.39.5[94]. Subsequently, the obtained high-quality sequence reads were clustered into ZOTUs by using USEARCH (https://www.drive5.com/usearch/).

To reveal potential contaminants introduced to the DHS reactor, we used 16S rRNA gene sequence information obtained from four drill mud samples of the IODP expedition (i.e., LMT159, LMT214, LMT27, and LMT372), which have been deposited in the GenBank Sequence Read Archive under accession numbers SRR1777632–SRR1777635[23]. The drilling mud sequence reads were cleaned up using the NGS QC Toolkit ver. 2.3[95] and then removed the PCR primers using Cutadapt v1.10[96] Taxonomic annotation was performed using QIIME. Because the tag sequence libraries of the drill mud samples were obtained using different PCR primers (V1-V3 regions) from the PCR primers used in the present study (V4-V5 regions), the OTUs having the same phylogenetic affiliation with OTUs from the drill mud samples were defined as the potential contaminants. In addition, we treated core members of the human microbiome[97] and members of the genus *Candidatus* Nitrosopumilus (an aerobic ammonium-oxidizing archaea)[98] as potential contaminants.

PCR amplification of 18S rRNA gene of reactor enrichment samples. To evaluate the microbial eukaryotic community in reactor enrichments, 18S rRNA gene targeting primer pairs EukA/EukB[99] and Euk1391F (5'-GTACACACCGCCCGTC-3', a variant of a primer reported by Amaral-Zettler et al., 2009[100])/EukB were used for PCR amplification. The conditions of PCR amplification followed the protocols described in a previous study[101] and The Earth Microbiome Project (http://www.earthmicrobiome.org/emp-standard-protocols/18s/).

Isolation of anaerobic microorganisms from the DHS bioreactor. To isolate anaerobic microorganisms grown in the DHS reactor, batch-type cultivation was performed in 50 ml serum vials containing 20 ml media under an atmosphere of N_2/CO_2 (80/20 [v/v]) without shaking. The basal medium was the same medium used for the DHS reactor cultivation without organic compounds and contained one of the following energy sources: (i) each of the organic substrates in the medium (i.e., butyrate, propionate, acetate, and yeast extract), (ii)

each of the methanogenic substrates, such as H_2/CO_2, formate, trimethylamine and methanol, and (iii) a substrate for homoacetogenesis; i.e., H_2/CO_2 plus 2-bromoethanesulfonate, which is an inhibitor of methanogenesis (Supplementary Table S8). A mixture of effluent and lignite particle samples that were collected at 694 days of operation was inoculated into each medium using a syringe with an 18-gauge needle (approximately 2 ml effluent and several lignite particles). The culture vessels were set up in duplicate for each substrate. The cultures were incubated at 40°C, 55°C or 80°C. Cell growth was determined by microscopic observation, methane-production and/or acid-production. Isolation of microorganisms from the enrichment cultures was conducted by serial dilution in liquid culture and/or roll-tubes. The purity of the isolate was routinely checked by microscopy. The serum vials were sealed with butyl rubber stoppers and aluminum crimp seals. To monitor anaerobic conditions in the media, resazurin was added to the medium as a redox indicator. The 16S rRNA gene sequences of pure cultures were determined as described previously[19].

References

88. Tandukar, M., Uemura, S., Ohashi, A. & Harada, H. Combining UASB and the ‘fourth generation’ down-flow hanging sponge reactor for municipal wastewater treatment. *Water Sci. Technol.* **53**, 209–218 (2006).
89. Ijiri, A. *et al.* Biogeochemical processes involving acetate in sub-seafloor sediments from the Bering Sea shelf break. *Org. Geochem.* **48**, 47–55 (2012).
90. Zhang, J., Kobert, K., Flouri, T. & Stamatakis, A. PEAR: a fast and accurate Illumina Paired-End reAd mergeR. *Bioinformatics* **30**, 614–620 (2014).
91. Caporaso, J.G. *et al.* QIIME allows analysis of high-throughput community sequencing data. *Nat. Meth.* **7**, 335–336 (2010).
92. Edgar, R. C. Search and clustering orders of magnitude faster than BLAST. *Bioinformatics* **26**, 2460–2461 (2010).
93. Chen, S. *et al.* AfterQC: automatic filtering, trimming, error removing and quality control for fastq data. *BMC bioinformatics* **18**, 80 (2017).
94. Schloss, P.D. *et al.* Introducing mothur: open-source, platform-independent, community-supported software for describing and comparing microbial communities. *Appl. Environ. Microbiol.* **75**, 7537–7541 (2009).
95. Patel, R.K. & Jain, M. NGS QC toolkit: a toolkit for quality control of next generation sequencing data. *PLoS One* **7**, e30619 (2012).
96. Martin, M. Cutadapt removes adapter sequences from high-throughput sequencing reads.

EMBnet j. **17**, 10–12 (2011).

97. Li, K., Bihan, M. & Methé, B.A. Analyses of the stability and core taxonomic memberships of the human microbiome. *PLoS One* **8**, e63139 (2013).

98. Nunoura, T. *et al*. Variance and potential niche separation of microbial communities in subseafloor sediments off Shimokita Peninsula, Japan. *Environ. Microbiol.* **18**, 1889–1906 (2016).

99. Marsh, T.L., Liu, W.-T., Forney, L.J. & Hans, C. Beginning a molecular analysis of the eukaryal community in activated sludge. *Water Sci. Technol.* **37**, 455–460 (1998).

100. Amaral-Zettler, L. A., McCliment, E. A., Ducklow, H. W. & Huse, S. M. A method for studying protistan diversity using massively parallel sequencing of V9 hypervariable regions of small-subunit ribosomal RNA genes. *PLoS One* **4**, e6372 (2009).

101. Matsunaga, K., Kubota, K. & Harada, H. Molecular diversity of eukaryotes in municipal wastewater treatment processes as revealed by 18S rRNA gene analysis. *Microbes Environ.* **29**, 401–407 (2014).

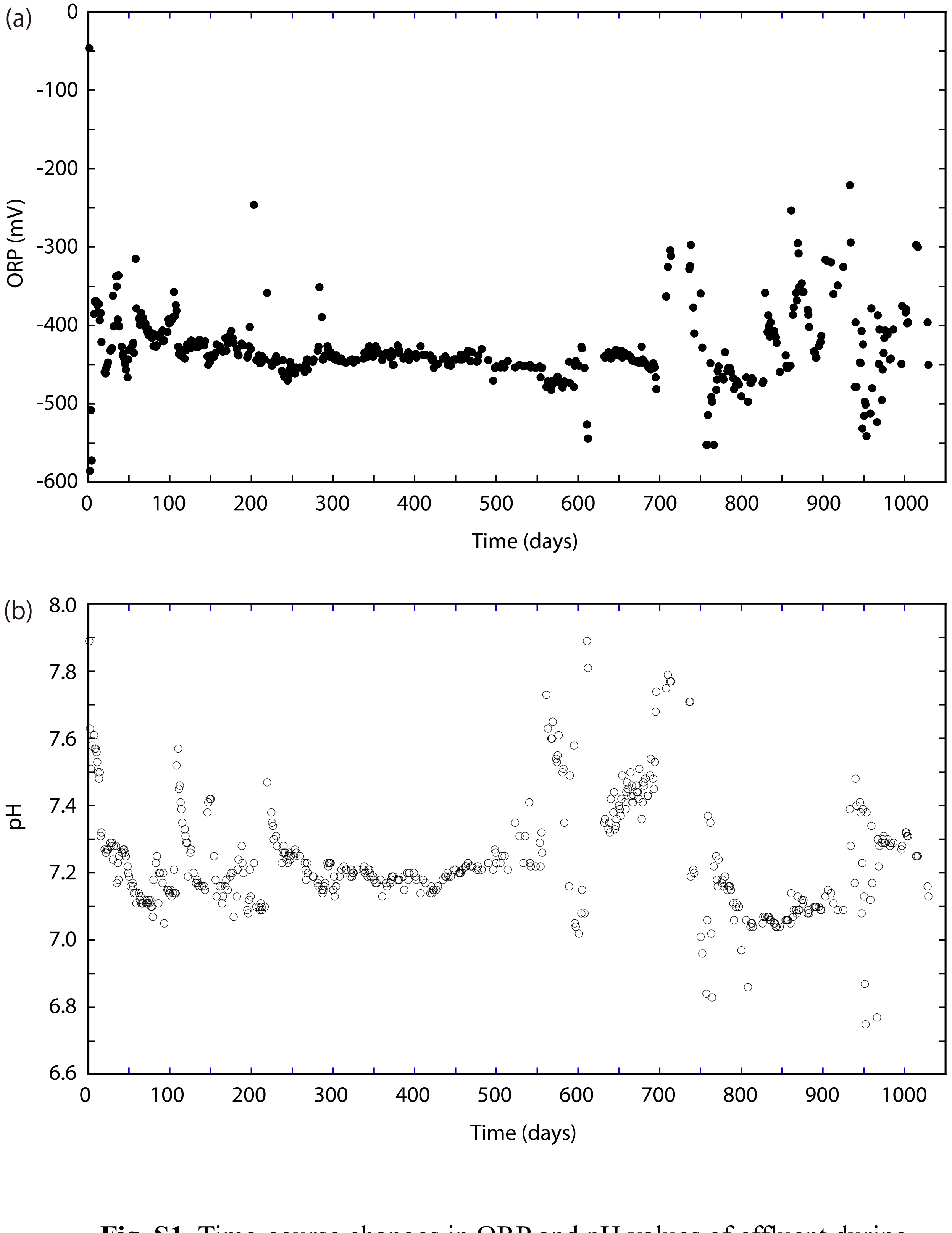

Fig. S1. Time-course changes in ORP and pH values of effluent during bioreactor operation. (a) ORP value. (b) pH value.

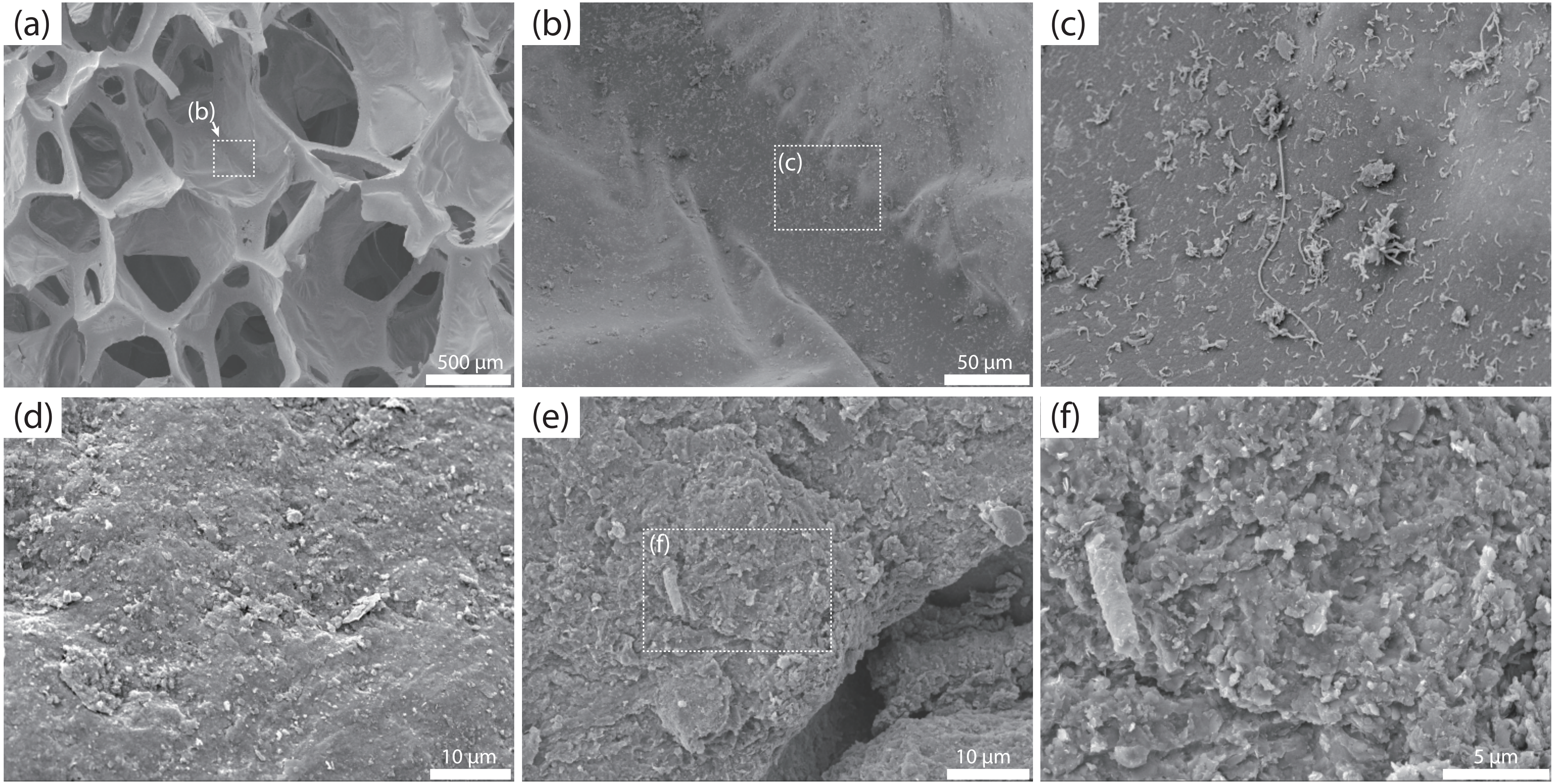

Fig. S2. Scanning electron micrographs of a polyurethane sponge carrier that was collected at 694 days of reactor operation and lignite particles before DHS reactor cultivation. (a-c) Polyurethane sponge carrier. (d) 15R-4 lignite sample. (e, f) 25R-5 lignite sample. White dotted-line squares indicate high-magnification areas.

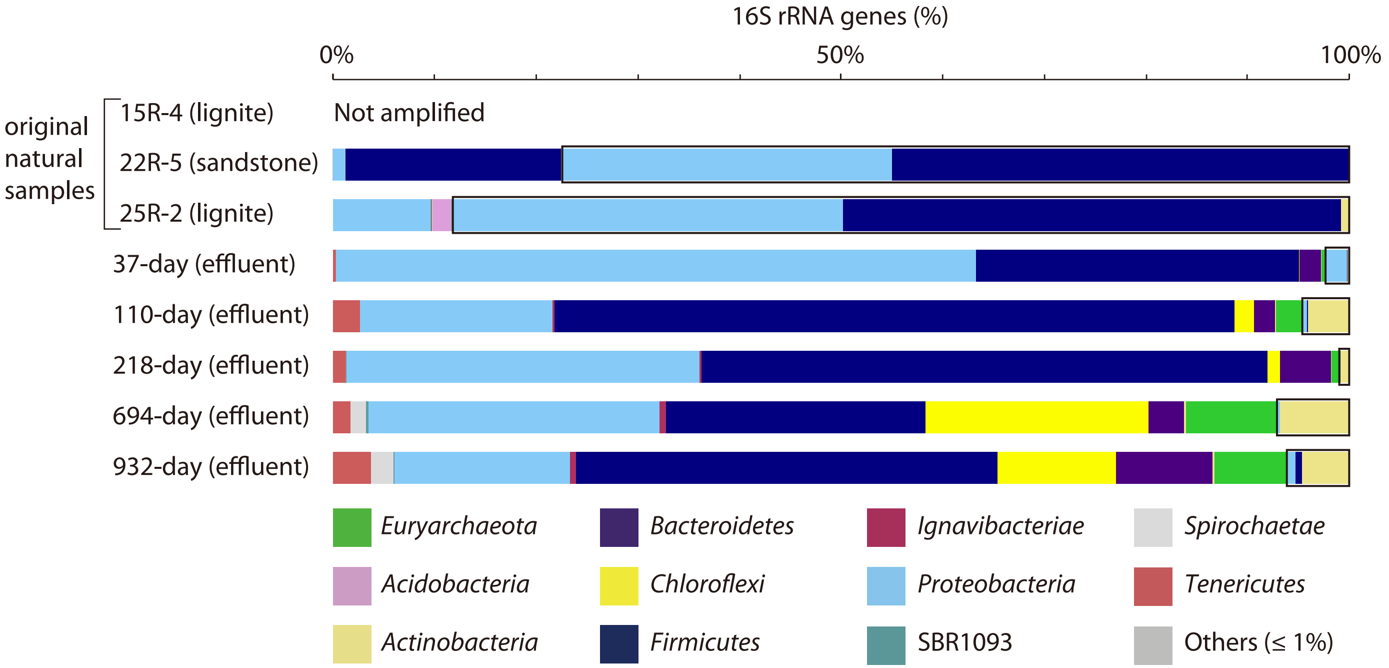

Fig. S3. Phylum-level taxonomic composition in original natural samples and effluent samples based on SSU rRNA gene-tag sequencing analyses. Black line boxes indicate potential contaminant populations. For the 15R-4 lignite sample, we performed PCR amplification three times using the extracted DNA. However, no PCR product was obtained.

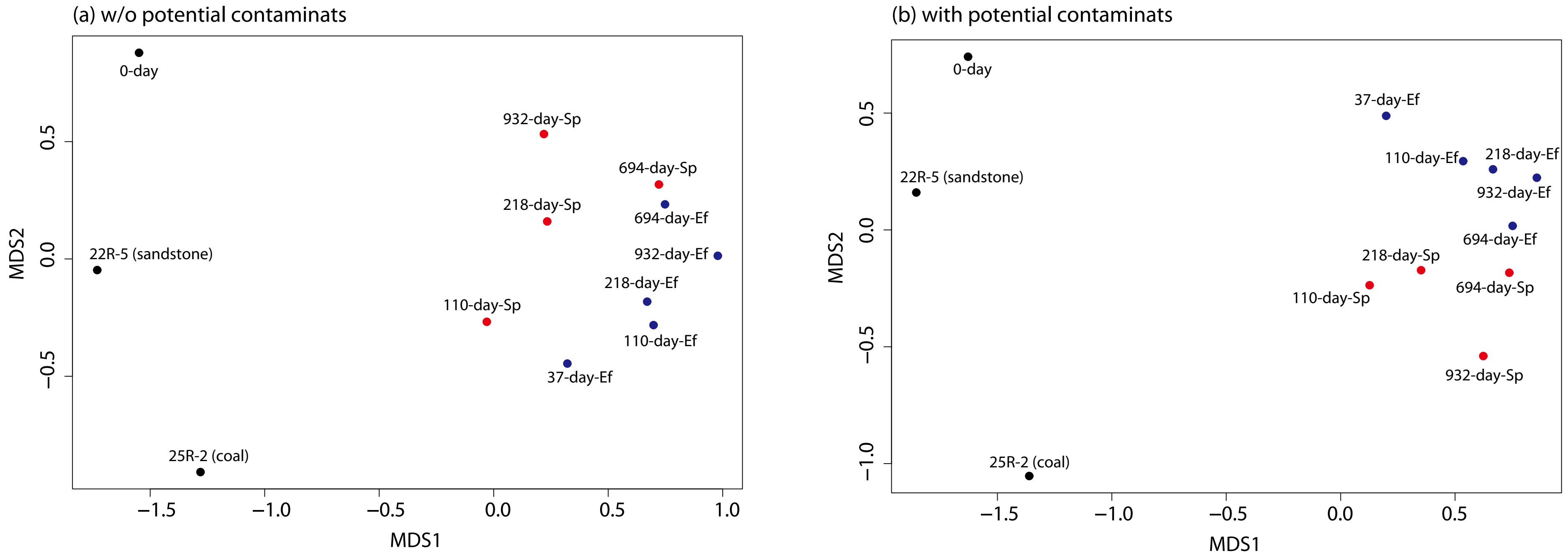

Fig. S4. Non-metric multidimensional scaling (NMDS) of the microbial community membership using the Bray-Curtis distance matrix. The name of each sample is composed of the sampling day (0, 37, 110, 218, 694, and 932 days) and an abbreviation of the sampling source (Sp, sponge carriers; Ef, effluent). (a) All sequences without potential contaminant sequences. (b) All sequences including potential contaminants.

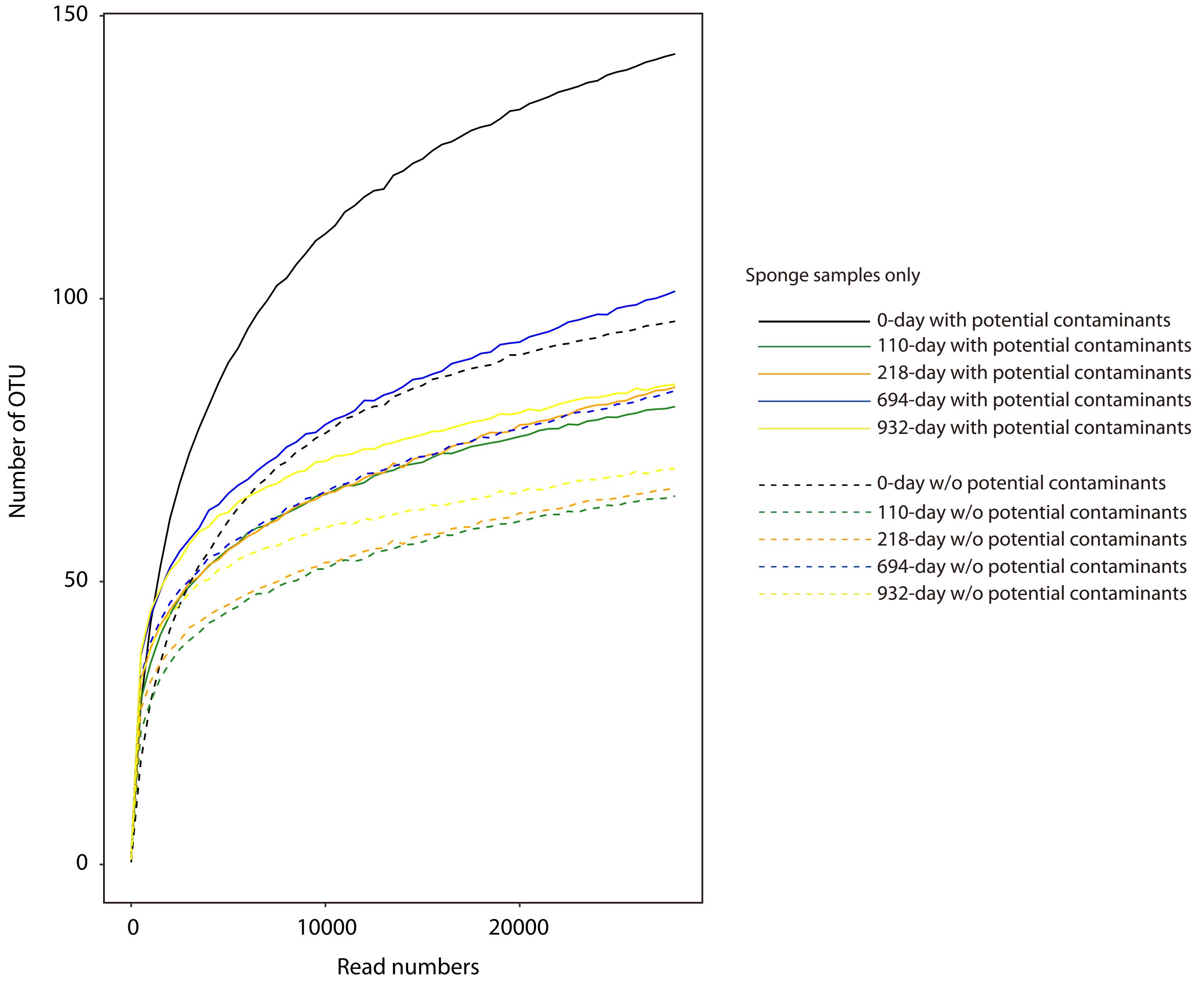

Fig. S5. Rarefaction curves representing the relationship between the number of sequences and the number of 97% OTUs identified.
